# High prevalence of fatigue in patients with inflammatory bowel disease: association with physical activity intensity and sedentary behavior

**DOI:** 10.3389/fpubh.2026.1893458

**Published:** 2026-08-27

**Authors:** María Dolores Herrera, Antoni Aguiló, Pedro Tauler, José Antonio Mingorance, Daniel Ginard, Eva Puchol, Sonia Martínez

**Affiliations:** 1 Gastroenterology Unit, Son Espases University Hospital, Palma, Balearic Islands, Spain; 2 Research Group on Evidence, Lifestyles and Health, Department of Nursing and Physiotherapy, Research Institute of Health Sciences (IUNICS), University of the Balearic Islands, Palma, Balearic Islands, Spain; 3 Health Research Institute of the Balearic Islands (IdISBa), University Hospital Son Espases, Palma, Balearic Islands, Spain; 4 Research Group on Evidence, Lifestyles and Health, Department of Fundamental Biology and Health Sciences, Research Institute of Health Sciences (IUNICS), University of the Balearic Islands, Palma, Balearic Islands, Spain

**Keywords:** fatigue, food avoidances, food supplements, inflammatory bowel diseases, physical activity intensity, remission, sedentarism

## Abstract

**Introduction:**

Fatigue is reported to be a common and disabling manifestation in patients with inflammatory bowel disease. Specialized units and nurse-led services play key roles in delivering holistic, long-term care for these patients. The aim of this study was to determine the prevalence of fatigue among inflammatory bowel disease patients attending a day hospital. The association between fatigue and physical activity was also analyzed, potentially providing clinically relevant information for nursing assessment and targeted interventions.

**Methods:**

A descriptive, cross-sectional study was performed on a sample of 422 patients (Crohn's disease, 64.5%; colitis, 35.5%) attending the day hospital of the Son Espases University Hospital (Palma, Balearic Islands, Spain). Sociodemographic and disease characteristics, physical activity levels, and sedentary time were determined using a questionnaire. Data were analyzed using descriptive statistics. The primary statistical analyses consisted of comparisons between participants with and without fatigue and across fatigue severity levels using Student's *t*-test, one-way ANOVA, or Pearson's chi-square test, as appropriate.

**Results:**

Most of the participants were in clinical remission (Crohn's disease, 95%; colitis, 71.1%). The prevalence of fatigue was 70.1%, with more than half of fatigued patients reporting a mild level of fatigue. Fatigue was found to be associated with depression and anxiety, with a higher prevalence of fatigue among the participants reporting to have these disorders (*p* = 0.010). Fatigue was associated with lower levels of physical activity (*p* = 0.002), mainly lower levels of intense physical activity, and in participants with severe fatigue. The participants with fatigue reported longer daily sitting times (*p* = 0.005). Furthermore, fatigue was associated with taking supplements (*p* = 0.016) and food avoidance (*p* < 0.001).

**Conclusions:**

A high prevalence of fatigue was found among patients with inflammatory bowel disease. Exercise intensity and fatigue level were found to be key factors, with fatigue being negatively associated with participation in physical activity. Patients with fatigue, particularly those with severe fatigue, engaged in lower levels of physical activity, especially high-intensity exercise. However, walking and moderate-intensity exercise appeared to be well tolerated. Additionally, fatigue was associated with being sedentary for greater durations.

## 1 Introduction

Inflammatory bowel disease (IBD) comprises a group of chronic, immune-mediated disorders of the gastrointestinal tract, including Crohn's disease (CD) and ulcerative colitis (UC). These conditions are characterized by chronic relapsing and remitting intestinal inflammation and commonly associated with abdominal pain, diarrhea, and rectal bleeding (1). Over recent decades, the incidence of IBD has increased substantially in industrialized countries, reaching 16.2 cases per 100,000 inhabitants per year in Spain in 2017, highlighting the growing burden it places on public health (2).

IBD-related symptoms may compromise patients' capacity to engage in exercise, sports, and leisure-time physical activities (3–5). Nevertheless, evidence regarding physical activity (PA) levels in individuals with IBD remains inconsistent, and current data do not conclusively demonstrate lower PA levels relative to the general population (5–7). In addition to PA, several lifestyle factors—including smoking, alcohol consumption, psychological stress, obesity, and dietary patterns—have been implicated in both the development and clinical course of IBD (8). Notably, smoking exerts differential effects according to disease subtype, being associated with worse outcomes in CD while not demonstrating the same detrimental impact in UC (8). Moreover, patients frequently report food intolerances linked to gastrointestinal symptoms (9, 10). Paradoxically, despite these dietary restrictions and symptom burdens, recent studies have documented a rising prevalence of overweight and obesity among individuals with IBD (8, 11, 12). Importantly, obesity has been associated with poorer clinical outcomes and increased disease complexity (13).

Beyond gastrointestinal manifestations, fatigue is increasingly recognized as a prevalent and disabling symptom in IBD (14, 15). Fatigue can be described as “a persistent, overwhelming sense of tiredness, weakness or exhaustion resulting in a decreased capacity for physical and/or mental work and is typically unrelieved by adequate sleep or rest” (16, 17). In IBD populations, fatigue has been associated with psychological comorbidities such as depression and anxiety, impaired quality of life, sleep disturbances, and obesity (18, 19). Its etiology is multifactorial; however, chronic systemic inflammation—a central feature of IBD —appears to play a pivotal role (20). Fatigue's prevalence has been reported to reach up to 86% in patients with active disease and range from 20 to 48% in those in clinical remission (19, 21), with higher prevalence consistently observed in women (22). Accordingly, the relationship between IBD severity and fatigue has become a major research focus, with differences according to sex also receiving attention (19, 21, 23). However, recent studies suggest that the prevalence of fatigue may be underestimated, particularly in patients in clinical remission.

PA may exert beneficial effects on fatigue through several mechanisms, including reducing systemic inflammation, preventing obesity, and improving cardiorespiratory fitness and sleep quality (24). However, most available studies have assessed overall PA levels in IBD without specifically addressing fatigue as an outcome. Furthermore, the relationship between PA intensity and fatigue has not been systematically examined, with limited evidence distinguishing between light, moderate, and vigorous activity. Sedentary behavior, recognized as an independent pro-inflammatory risk factor, has also been insufficiently investigated in relation to IBD-associated fatigue.

The aim of this study was to determine the prevalence of fatigue among patients with IBD attending a day hospital. Additionally, we analyzed lifestyle characteristics and explored the association between fatigue and lifestyle factors, particularly PA and sedentary behavior. Based on previous evidence, we hypothesized that there would be a high prevalence of fatigue and anticipated that fatigue would be associated with lower levels of PA as well as with adverse clinical and psychosocial outcomes such as depression and obesity.

## 2 Methods

### 2.1 Study design and participants

A descriptive cross-sectional study was performed on a sample of patients attending the Day Hospital for Inflammatory Bowel Disease of the Son Espases University Hospital (Palma, Balearic Islands, Spain) between January 2023 and March 2024. The inclusion criteria were being more than 18 years old, having a confirmed diagnosis of CD or UC, having attended the IBD Day Hospital during the recruitment period, and having provided written informed consent. No exclusion criteria were established, and all patients attending this unit during the period indicated above were invited to participate in the study (*n* = 435). Among them, 13 patients declined to participate. Thus, the final number of participants was 422, comprising 214 women (50.7%) and 208 men (49.3%).

All participants were informed of the purpose and requirements of the study and signed the written consent form to participate. The protocol aligned with the Declaration of Helsinki for research involving human subjects and was approved by the Research Ethics Committee of the Balearic Islands (CEI-IB Ref: IB 4993/22 PI).

### 2.2 Data collection

The following data were collected from the participants using a survey administered through a paper-and-pencil interviewing method (face-to-face). Items derived from validated instruments were retained in their original form, whereas study-specific descriptive questions were designed *a priori* for this investigation. The responses were coded using predefined categories, and numerical codes were assigned to each answer option before database entry. The full questionnaire is provided in the Supplementary material.

- Sociodemographic variables: Information on gender and age was collected.
- Anthropometrical measurements: Mass and stature were measured, and body mass index (BMI) was calculated. The participants were categorized depending on their BMI in accordance with the following World Health Organization (WHO) criteria: underweight (BMI < 18.5 kg/m^2^), normal weight (BMI = 18.5–24.9 kg/m^2^), overweight (BMI = 25.0–29.9 kg/m^2^), and obese (BMI ≥ 30.0 kg/m^2^) (25).
- Fatigue: The Spanish version of the Functional Assessment of Chronic Illness Therapy-Fatigue questionnaire (FACIT-F), previously validated in Spanish for IBD patients, was used to determine whether a patient had fatigue (26). This questionnaire consists of 13 questions, each presented with a 5-point Likert scale (0—not at all; 4—very much), and the cut-off score was set at 45 points, with higher scores revealing absence of fatigue. Furthermore, the following fatigue levels were considered: mild (31–44 points), moderate (21–30 points), and severe (0–20 points). As previously reported, a difference of 3–4 units is considered the minimal clinically important difference (26).
- Physical activity levels: The validated standard short-form of the International Physical Activity Questionnaire (IPAQ) was used to determine participant PA assessed in metabolic equivalents (METs) per hour per week (27). The participants were also categorized into high-, moderate-, and low-PA-level groups. Total weekly PA time and daily sitting time were also determined.
- Food avoidance: Food avoidance was ascertained using the following investigator-developed open question: indicate which foods you do not eat except for those excluded by your type of diet. Responses were recorded verbatim by the interviewer and subsequently coded independently by two members of the research team into predefined food categories: fiber-rich foods (legumes, whole grain foods, etc.); vegetables; fish and shellfish/seafoods; red meats and processed foods; high-protein foods (non-red meat and eggs); spices, herbs, and condiments; fruits; dairy products; starchy foods; non-essential foods/discretionary foods (sugar, chocolate, etc.); beverages (soft drinks, energy drinks, fruit juices, and alcoholic drinks); nuts; and others.
- Dietary supplements: The participants were asked whether they consumed any dietary supplements (Do you take any dietary supplements? YES/NO). Those answering affirmatively were subsequently asked to specify the supplements consumed. The responses were recorded verbatim and coded into predefined categories including vitamins, minerals, omega-3 fatty acids, protein supplements, probiotics, collagen, and other supplements.
- Clinical data: Type of IBD (CD or UC), localization and extension of the disease, duration of disease, previous surgery, and treatment were collected from clinical records. IBD clinical disease activity was assessed using the Harvey–Bradshaw Index for CD and the Mayo Clinic Index for UC (28). Active disease was defined as a Harvey–Bradshaw Index score ≥5 for CD or a Mayo Clinic Index score >0 for UC. Affliction with other active chronic diseases, including depression and anxiety, was recorded from the participants' clinical records.
- Pharmacological treatments: Whether the participants were undergoing any pharmacological treatments, specifically biological treatments, was ascertained. This information was obtained from clinical records.
- Alcohol consumption: Frequency of alcohol consumption was ascertained using the following question from the Alcohol Use Disorders Identification Test (AUDIT): “How often do you drink alcoholic beverages?” (answers: never, once a month or less, 2–4 times a month, 2–3 times per week, or 4 or more times per week) (29).
- Smoking habits: The participants were asked if they smoked (YES/NO)—in reference to tobacco consumption and cigarette use—and then classified as non-smokers, ex-smokers, occasional smokers, or (daily) smokers, according to the criteria of the 2020 European Health Survey in Spain (30).
- Hematological parameters: Hemoglobin, iron, and ferritin concentrations previously measured during a scheduled routine visit to the day hospital were recorded. As per the WHO definition, anemia was defined as a hemoglobin level < 13 g/dL for males and < 12 g/dL for females (29).

### 2.3 Statistical analysis

Statistical analysis was performed using IBM SPSS Statistics 23.0 software (IBM, Chicago, IL, USA). Statistical significance was set at a *p*-value below 0.05. All the data were tested for normal distribution (Kolmogorov–Smirnov test). Descriptive analysis was used to report the frequencies and percentages of categorical variables, and the mean and standard deviation (SD) were reported for quantitative variables. Either Student's *t*-test for unpaired data, the one-way ANOVA, or the Pearson's chi-square (χ^2^) test was used to evaluate differences between groups.

## 3 Results

### 3.1 Characteristics of participants in the study

Table 1 shows the characteristics of the study participants and the values for measured variables. In total, 64.5% of the participants in the study presented CD, while 35.5% presented UC, with no differences between genders (*p* = 0.231). Most patients were in clinical remission, and no differences between genders were observed for severity indices (CD: *p* = 0.155; UC: *p* = 0.946). Furthermore, most participants (94.8%) were receiving pharmacological treatment, with 80.6% receiving biologic therapy. The most frequently prescribed biologic agents were ustekinumab (29.1%), infliximab (21.8%), and adalimumab (15.9%). Among non-biologic therapies, oral mesalazine was the most commonly used treatment (20.4%), followed by azathioprine (2.8%) and topical mesalazine (1.9%).

**Table 1:** Characteristics of participants in the study, stratified per gender.

	All (*n* = 422)	Women (*n* = 214)	Men (*n* = 208)	*p*
Age (years)	44.9 ± 15.0	44.6 ± 14.8	45.3 ± 15.0	0.653
BMI				< 0.001^*^
Underweight	18 (4.3)	13 (6.1)	5 (2.4)	
Normal weight	219 (51.7)	127 (59.2)	92 (44.2)	
Overweight	133 (31.5)	48 (22.5)	85 (40.9)	
Obesity	52 (12.5)	26 (12.2)	26 (12.5)	
IBD				0.231
Crohn's disease	272 (64.5)	144 (67.1)	128 (61.5)	
Ulcerative colitis	150 (35.5)	70 (32.9)	80 (38.5)	
IBD severity				
Crohn's disease (Harvey)	0.94 ± 1.77	1.09 ± 1.88	0.78 ± 1.65	0.155
Ulcerative colitis (Mayo)	0.74 ± 1.48	0.74 ± 1.55	0.76 ± 1.44	0.946
Clinical remission				
Crohn's disease	260 (95.5)	137 (95.0)	123 (96.0)	0.853
Ulcerative colitis	107 (71.1)	51 (72.2)	56 (70.0)	0.717
Previous IBD surgery	85 (20.1)	48 (22.5)	37 (17.8)	0.295
Pharmacological treatment	400 (94.8)	205 (95.8)	195 (93.8)	0.351
Biological treatment	341 (80.6)	176 (82.2)	165 (79.3)	0.461
Depression/anxiety	20 (4.8)	16 (7.5)	4 (1.9)	0.007^*^
Other diseases	155 (36.7)	80 (37.4)	75 (36.1)	0.712
Blood parameters				
Hemoglobin (g/dL)	14.0 ± 1.7	13.5 ± 1.7	14.6 ± 1.6	< 0.001^*^
Iron (mg/dL)	82.3 ± 35.9	82.2 ± 37.3	82.7 ± 34.5	0.888
Ferritin (ng/mL)	97.5 ± 130.5	75.8 ± 98.3	120.1 ± 153.9	0.001^*^

Values are expressed as mean ± standard deviation, or number of participants (percentage). ^*^p < 0.05 indicates significant differences between genders (Student's t test for unpaired data or chi-square test).

BMI, body mass index; IBD, inflammatory bowel disease; CD, Crohn's disease; UC, ulcerative colitis.

The prevalence of overweight and obesity was 31.5% and 12.6%, with higher figures in men than in women (*p* < 0.001). A higher prevalence of depression and anxiety was found in women than in men (*p* = 0.007). Hemoglobin (*p* < 0.001) and ferritin (*p* = 0.001) concentrations were higher in men than in women, although both remained within the recommended reference ranges, which are higher for men.

**Figure 1:**
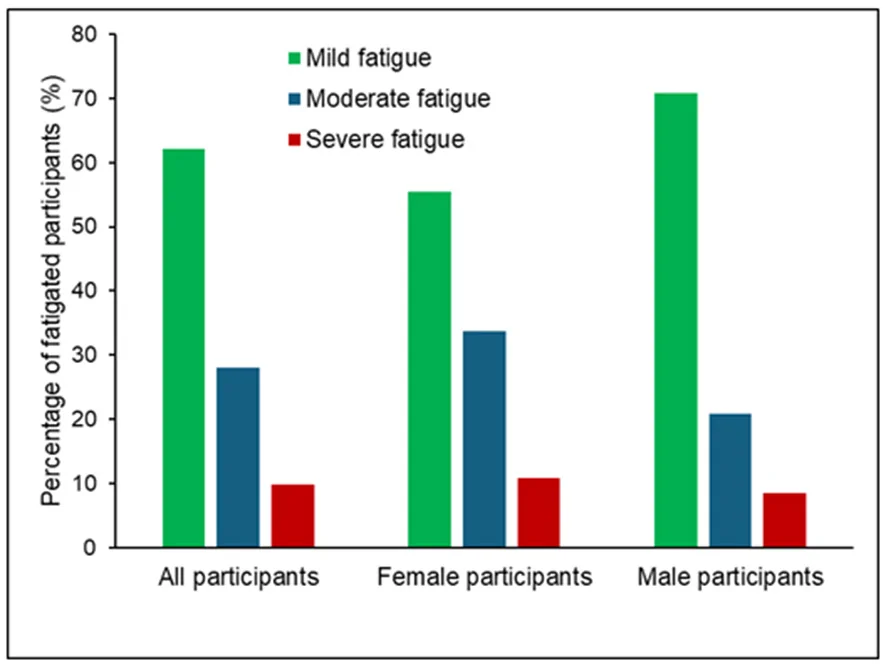
Fatigue levels in fatigued participants stratified per gender.

The prevalence of fatigue among the participants in the study was 70.1%, with statistically significantly higher values in women than in men (77.8 vs. 62.5%, *p* = 0.001). A mild level of fatigue was found in more than half of the fatigued participants (62.2%), with higher percentages of moderate and severe fatigue levels in women than in men (*p* = 0.023, Figure 1).

### 3.2 Participant lifestyles

Table 2 reports parameters related to the participants' PA levels. In total, 65.9% of the participants could be considered active, with a higher percentage for men (*p* < 0.001). Male participants engaged in more PA, and they also had higher figures for the frequency and duration of high-intensity PA bouts.

**Table 2:** Lifestyles in participants stratified per gender.

	All (*n* = 422)	Women (*n* = 214)	Men (*n* = 208)	*p*
IPAQ levels				< 0.001^*^
Low	144 (34.1)	89 (41.6)	55 (26.4)	
Moderate	96 (22.8)	57 (26.5)	39 (18.8)	
High	182 (43.1)	68 (31.9)	114 (54.8)	
Total PA				
Mets·hour/week	39.9 ± 70.1	32.0 ± 54.6	48.2 ± 59.8	0.004^*^
Sessions/week	8.92 ± 4.36	8.19 ± 4.01	9.69 ± 4.57	< 0.001^*^
Minutes/day	209.4 ± 203.1	193.8 ± 195.6	225.4 ± 209.8	0.111
Intense PA				
Sessions/week	1.44 ± 1.99	0.93 ± 1.61	1.97 ± 2.19	< 0.001^*^
Minutes/day	39.4 ± 70.1	27.0 ± 60.9	52.0 ± 76.7	< 0.001^*^
Moderate PA				
Sessions/week	2.30 ± 2.39	2.16 ± 2.28	2.44 ± 2.50	0.218
Minutes/day	62.3 ± 90.6	59.3 ± 93.8	65.3 ± 87.6	0.497
Walking				
Sessions/week	5.18 ± 2.36	5.11 ± 2.39	5.28 ± 2.30	0.446
Minutes/day	107.7 ± 132.6	107.8 ± 134.0	108.1 ± 131.5	0.984
Sitting time				
Hours/day	6.21 ± 3.37	5.99 ± 3.15	6.42 ± 3.57	0.193

Values are expressed as mean ± standard deviation or number of participants (percentage); ^*^p < 0.05 indicates significant differences between genders (Student's t test for unpaired data or chi-square test).

PA, physical activity; IPAQ, international physical activity questionnaire; Mets, metabolic equivalents.

Supplementary Table 1 reports on smoking and alcohol consumption among the participants. Male participants reported consuming alcohol more often than female participants (*p* < 0.001), while female participants reported higher percentages of food avoidance (*p* = 0.002). A total of 37.0% of the participants were ex-smokers, and no differences between genders were observed in terms of smoking habits (*p* = 0.599). However, there was a higher percentage of daily smokers among patients with CD than among patients with UC (21.0 vs. 4.0%, *p* < 0.001).

In total, 19.9% of the participants reported taking at least one dietary supplement, with no differences between women and men (20.7 vs. 18.8%, *p* = 0.623). The most common supplements taken were vitamins (with 50% of the participants having reported taking any vitamin supplement), minerals (27.4%), omega-fatty acids (15.5%), proteins (14.3%), probiotics (13.1%), and collagen (10.7%). Overall, 56.2% of the participants reported food avoidance, with no differences between diseases (*p* = 0.091) and severity of the disease (*p* = 0.423) but with a higher prevalence in women (57.4 vs. 42.6%, *p* = 0.002). The highest prevalences of avoidance were observed for vegetables (with 34.2% of the participants having reported any avoidance), fiber (28.7%), dairy products (26.6%), fruit (18.6%), spicy (13.9%), and red meat (13.5%). A higher prevalence of vegetable avoidance was observed among patients with CD relative to those with UC (39.8 vs. 22.4%, *p* = 0.008).

### 3.3 Association between fatigue and participants' characteristics

Associations between fatigue and parameters such as age, BMI, or type of IBD were found to be non-significant (Table 3 and Supplementary Table 2). Bivariate correlation analysis showed that FACIT-F values were inversely correlated with the severity of the disease (CD: Pearson = −0.260, *p* < 0.001; UC: Pearson = −0.236, *p* = 0.003). This association was also observed for CD when different fatigue levels were considered (*p* = 0.003). However, fatigue was not associated with the location of the IBD.

**Table 3:** Association of fatigue with characteristics of participants.

	No fatigue (*n* = 126)	Fatigue (*n* = 296)	*p*
Age (years)	43.5 ± 15.5	45.5 ± 14.8	0.220
BMI			0.111
Underweight (%)	3 (2.4)	15 (5.1)	
Normal weight (%)	67 (53.2)	151 (51.0)	
Overweight (%)	46 (36.5)	87 (29.4)	
Obesity (%)	10 (7.9)	43 (14.5)	
IBD			0.475
Crohn's disease	78 (28.7)	194 (71.3)	
Ulcerative colitis	48 (32.0)	102 (68.0)	
Disease scores			
Crohn's disease (Harvey)	0.58 ± 1.34	1.08 ± 1.90	0.044^*^
Ulcerative colitis (Mayo)	0.46 ± 1.11	0.88 ± 1.61	0.098
Previous IBD related surgery	18 (14.3)	67 (22.6)	0.050
Depression/anxiety	1 (0.8)	20 (6.8)	0.010^*^
Other diseases	38 (30.2)	109 (36.8)	0.188
Blood parameters			
Hemoglobin (g/dL)	14.5 ± 1.5	13.8 ± 1.8	< 0.001^*^
Iron (μg/dL)	89.4 ± 35.4	79.3 ± 35.9	0.010^*^
Ferritin (ng/mL)	91.9 ± 96.8	99.9 ± 142.6	0.570

Values are expressed as mean ± standard deviation or number of participants (percentage); ^*^p < 0.05 indicates significant differences (Student's t test for unpaired data or chi-square test).

IBD, inflammatory bowel disease; BMI, body mass index.

Fatigue was also found to be associated with affliction with depression and anxiety, with a higher prevalence of fatigue among participants who reported having these disorders (*p* = 0.010 for affliction with fatigue, and *p* = 0.036 when fatigue levels were considered). Significantly lower scores on the FACIT-F questionnaire, indicating higher fatigue, were observed among patients with depression (30.6 ± 9.4 vs. 37.6 ± 10.5; *p* = 0.003). Lower values of hemoglobin (*p* = 0.020) and iron (*p* = 0.010) were found in patients with fatigue.

### 3.4 Association between fatigue and lifestyle

Fatigue was associated with lower levels of PA [*p* = 0.002 for affliction with fatigue, and *p* < 0.001 when fatigue levels were considered (Table 4 and Supplementary Table 3)]. These differences basically manifested when severe fatigue levels and intense PA parameters were considered. Notably, the participants with fatigue reported longer daily sitting times (*p* = 0.005 for affliction with fatigue, and *p* = 0.023 when fatigue levels were considered).

**Table 4:** Association of fatigue with physical activity parameters.

	No fatigue (*n* = 126)	Fatigue (*n* = 296)	*p*
IPAQ levels			0.002^*^
Low (%)	23 (18.3)	121 (40.9)	
Moderate (%)	34 (27.0)	62 (20.9)	
High (%)	69 (54.8)	113 (38.2)	
Total PA			
Mets·hour/week	43.6 ± 47.3	38.4 ± 61.6	0.404
Sessions/week	10.0 ± 4.4	8.4 ± 4.3	0.001^*^
Minutes/day	222.21 ± 88.7	204.42 ± 09.2	0.412
Intense PA			
Sessions/week	1.93 ± 2.19	1.24 ± 1.86	0.002^*^
Minutes/day	42.3 ±49.7	38.3 ± 77.2	0.594
Moderate PA			
Sessions/week	2.66 ± 2.57	2.14 ±2.29	0.053
Minutes/day	61.9 ± 81.1	62.4 ± 94.6	0.958
Walking			
Sessions/week	5.44 ±2.22	5.07 ±2.41	0.139
Minutes/day	118.01 ± 33.1	103.71 ± 32.4	0.312
Sitting time			
Hours/day	5.50 ± 3.04	6.51 ± 3.47	0.005^*^

Values are the mean ± SD or number of participants (percentage); ^*^p < 0.05 indicates significant differences (Student's t test for unpaired data or chi-square test).

Non-significant associations were found between fatigue and smoking or alcohol consumption (Supplementary Table 4). However, a higher percentage of participants with fatigue reported taking supplements (23 vs. 12.7%, *p* = 0.016). Furthermore, fatigued patients reported a higher prevalence of food avoidance (61.8 vs. 42.9%, *p* < 0.001). These results refer to overall dietary supplement use and overall food avoidance (yes/no), rather than to specific types of supplements or food groups.

## 4 Discussion

This study was conducted using a cohort of patients with IBD attending a day hospital, implying that most of the participants were undergoing biological therapy while in clinical remission. Despite these conditions, a high prevalence of fatigue was observed among the participants. Furthermore, fatigue was associated with lower vigorous PA levels, intake of dietary supplements, and affliction with depression or anxiety.

In agreement with previous studies, the prevalence of fatigue among patients with IBD—even among those in clinical remission—was high, with values up to sevenfold higher than those reported in the general population, in which prevalence is typically below 10% (23, 31, 32). Considering the high proportion of participants in clinical remission, the observed prevalence of fatigue was higher than that reported in previous studies (19, 21) and more closely resembled figures described for patients with active disease (23). This prevalence substantially exceeds the rates previously reported in patients in clinical remission (typically 20–48%), suggesting that the burden of fatigue in IBD may be underrecognized in routine clinical practice. However, it has also been suggested that the lack of consensus regarding the definition of fatigue may influence its assessment and hinder comparability across studies, particularly when different measurement tools (e.g., distinct questionnaires or cut-off values) are used (14, 15, 22).

Within this overall high prevalence, a higher prevalence of fatigue was observed among women, particularly at moderate and severe levels, whereas the proportion of mild fatigue was similar between sexes. In a previous study (19), sex differences in fatigue prevalence were identified in univariate analyses but not in multivariate models, suggesting that the observed differences may be explained by confounding factors that are more prevalent or severe in women, such as depression or sleep disturbances (33, 34). These conditions are closely interrelated and strongly associated with fatigue (34, 35). Indeed, fatigue has frequently been described as exhibiting a female predominance across a wide range of disorders, including depression, as well as in the general population (32, 36). In this study, fatigue was significantly associated with depression, which may partly explain the observed sex differences, given the significantly higher prevalence of depression among women. Although the underlying mechanisms remain unclear, fatigue in IBD has consistently been associated with anxiety and depressive disorders (18, 19). Moreover, it has been suggested that psychological comorbidities may contribute more substantially to disability and functional impairment in patients with IBD than gastrointestinal symptoms themselves (37).

The potential association between IBD severity and fatigue has been widely investigated, with inconsistent findings reported across studies (19, 23). In this study, a statistically significant, albeit weak, correlation was found between disease severity and fatigue scores. However, the difference in mean fatigue scores between patients in clinical remission and those with active disease did not reach statistical significance despite being close to the threshold of clinical relevance (37.6 vs. 34.8). Therefore, a relationship between disease severity and fatigue could not be definitively established. The high prevalence of fatigue among patients in clinical remission has been proposed as a possible explanation for the lack of a clear association between fatigue and disease activity observed in previous research (38, 39).

Anemia is among the most frequent extraintestinal complications of IBD and may partially explain the association between disease severity and fatigue (19, 38). In this study, the prevalence of anemia was lower than that in previous studies reporting prevalences of up to 26% (19), which could be attributed, at least in part, to the different disease severities of the participants considered. Furthermore, and in agreement with previous studies, no statistically significant association was found between anemia and fatigue (19, 38). This finding has been explained by the slow development of anemia commonly observed in these patients, allowing them to adapt to low levels of hemoglobin and develop tolerance to the symptoms of anemia (19). Furthermore, in this study, most patients with anemia, around 80%, had hemoglobin concentrations higher than 11 g/dL, indicating mild-severity anemia (40). Taken together, these observations could indicate a limited effect of anemia on self-perception of fatigue.

Although no significant associations were observed between fatigue and obesity, the high prevalences of obesity and overweight identified in this cohort are noteworthy. Overweight and obesity in IBD patients have been associated with more-severe hospitalizations and negative clinical outcomes, a greater risk of active disease, and an earlier loss of response to therapy (8). The issue of obesity is becoming increasingly prominent within the context of IBD, as it was historically underrecognized due to the frequent association between IBD and malnutrition (41). The rates of adult obesity among these patients are 15–40%, and an additional 20–40% of patients are overweight, with no difference between CD and UC (8, 11, 12). Similar figures regarding overweight were observed in our population, although obesity rates were slightly lower. This high prevalence of obesity could be independent of the underlying IBD and has been associated with smoking cessation, corticosteroid use, and the use of biological agents (42). In this regard, in this study, most of the participants were undergoing biological treatment, and the highest prevalence of overweight and obesity was observed among former smokers (results not shown), who, in fact, made up a higher proportion than usual. Therefore, this finding may support the notion that quitting smoking contributes to increasing the prevalence of overweight and obesity.

Regarding smoking habits, in addition to the high proportion of ex-smokers observed, our findings are in line with previous reports showing a persistently high prevalence of current smokers among patients with CD (43, 44), among whom smoking was five times more prevalent relative to UC patients. Furthermore, previous studies have demonstrated that many of these patients are unaware of the detrimental effects of smoking on CD course and outcomes (43, 44). Cigarette smoking has been associated with increased intestinal inflammation and oxidative stress, mechanisms that may contribute to disease activity (8). In this regard, cigarette smoking has been associated with an increased risk of developing CD (45) as well as with a more severe phenotype, complicated behavior, and adverse outcomes such as an ileum-dominant CD, perianal disease, and a stricturing phenotype (8). Unlike CD, smoking does not negatively impact clinical outcomes in patients with established UC and may be protective against adverse outcomes (45). These results could support the integration of smoking cessation strategies into routine IBD care, particularly for individuals with CD.

Development of IBD, particularly the active symptoms such as abdominal pain or fecal incontinence, may impair patients' ability to engage in exercise and participate in sports activities (3–5), potentially contributing to overweight and obesity. However, other studies have reported comparable PA levels between patients with quiescent IBD and healthy controls (5–7). In this study, PA levels measured in non-fatigued patients were very similar to those previously reported by our group in a healthy and younger population (43.6 vs. 43.8 METs·hour·week^−1^) (46). Notably, assessment of not only the presence of fatigue but also its severity allowed us to identify differences in the association between PA and fatigue across fatigue levels. In this regard, the PA pattern of patients with mild fatigue more closely resembles that of non-fatigued patients than that observed in individuals with moderate or severe fatigue. In contrast, lower PA levels were evident among patients with moderate and severe fatigue. These findings support the notion that there is a strong association between PA intensity and fatigue severity. Differences between fatigued and non-fatigued patients were primarily observed in regard to vigorous-intensity PA, with lower levels in fatigued individuals. However, no statistically significant differences were found in terms of time spent walking, and differences in moderate exercise became evident only when patients with severe fatigue levels were considered. Previous studies have similarly reported that fatigued patients engage in lower-intensity PA and exhibit lower physical fitness relative to non-fatigued patients (47). It has also been observed that fatigued individuals with IBD tend to prefer walking and moderate-intensity exercise over vigorous activities (5). In this context, repeated prolonged moderate-intensity walking has been shown to elicit similar cytokine responses in individuals with and without this condition and does not appear to affect fecal calprotectin concentrations, suggesting that exercise at this intensity is safe for patients with this disease (48). Taken together, these findings indicate that moderate-intensity exercise is generally well tolerated, even in the presence of fatigue, whereas a reduced capacity to perform vigorous-intensity exercise may be a sensitive indicator of fatigue severity.

At the same time, notably, fatigued patients reported more time spent sedentary relative to non-fatigued patients. Although sedentary time was assessed using a single question from the IPAQ, this variable was not routinely evaluated in previous studies examining fatigue in patients with IBD. Prolonged sedentary time may also reflect core features of fatigue, such as persistent tiredness, low energy, or a sense of exhaustion (16, 17). Therefore, the identification of sedentary time as an independent factor associated with fatigue represents a relatively unexplored dimension in IBD research. This finding is particularly relevant given the well-established role of sedentary behavior as an independent contributor to systemic inflammation (49, 50). This association indicates the possibility of the existence of a positive feedback loop in which fatigue promotes sedentary behavior, which in turn may exacerbate inflammation and further aggravate fatigue, thereby generating a self-perpetuating cycle. Conversely, regular physical activity has well-established anti-inflammatory effects, mediated by mechanisms such as the release of anti-inflammatory myokines, improved immune regulation, and reductions in adipose-tissue-derived inflammatory mediators (51, 52). In patients with IBD, moderate-intensity exercise appears to be safe and does not exacerbate intestinal inflammation (48). Although causality cannot be inferred from our findings, the lower physical activity levels observed among fatigued patients may reduce exposure to these beneficial anti-inflammatory effects, potentially contributing to the continuance of fatigue.

Food avoidance is commonly reported by patients with IBD because, among other reasons, patients feel that there are strong links between diet and triggering flare-ups and maintaining remission (9, 10, 51). In agreement with previous results, in our study, the occurrence of food avoidance was similar in patients with CD and UC, with comparable patterns in both diseases (9). Consumption of foods with higher fiber content, such as vegetables, fruits, and legumes (9), as well as dairy products (9, 10, 52), was reported more often by the participants. While dairy products have consistently been pointed out in similar studies (9, 10, 51), fiber-rich products have not always been associated with food intolerances in patients with IBD (10). The inconsistency in the results concerning specific food groups, combined with the generally non-significant associations with disease characteristics such as IBD subtype, anatomical location, or clinical severity, has given rise to the hypothesis that such food intolerances may not be pathogenetically driven (9, 10). Rather, they could reflect food groups known to elicit gastrointestinal symptoms even in individuals without an underlying pathology (9). It is also noteworthy that, in this study, fatigued patients reported higher prevalences of both self-reported nutritional supplement use and food avoidance. In this regard, some previous studies have documented a higher prevalence of self-reported food intolerance in patients with chronic fatigue (53–55). At the same time, dietary interventions, such as elimination diets excluding gluten, dairy, and additives, have been associated with symptomatic improvements in fatigue and gastrointestinal distress among patients with chronic fatigue (56). However, the underlying mechanisms and causal relationship remain unclear, and it has been suggested that food intolerance could be one manifestation of the somatization trait expressed in patients with fatigue (54).

Avoidance of specific foods and beverages could explain, at least in part, the commonality of supplement consumption among patients with IBD, a practice that has been associated with an insufficient intake of micronutrients (5). In this study, approximately one in five participants reported using dietary supplements, with no significant differences according to sex or disease but with a significant trend toward higher consumption among fatigued patients. The higher prevalence of supplement use among fatigued patients should not be interpreted as evidence that supplements contribute to fatigue. Rather, patients experiencing fatigue may be more likely to try dietary supplements on their own or receive advice regarding supplement use in an attempt to alleviate their symptoms. This should not be interpreted as a nutritional intervention or a comprehensive dietary management strategy. Therefore, supplement use is more likely to represent a consequence or marker of fatigue rather than its cause. The most frequently reported supplements were vitamins, minerals, protein preparations, omega fatty acids, and probiotics. Micronutrients such as vitamin D, vitamin B12, and folate, as well as adequate protein intake, play essential roles in immune regulation and maintenance of gut integrity and have been associated with improved clinical outcomes and disease remission (57, 58). Probiotics, prebiotics, and dietary strategies aimed at modulating gut microbiota composition have shown potential benefits regarding managing symptoms and reducing inflammation (57). Therefore, optimizing gut microbiota composition and ensuring adequate vitamin and protein intake represent important components of IBD management and may help explain the relatively high prevalence of dietary supplement use among IBD patients (10).

This study has several limitations that should be acknowledged. First, its cross-sectional design precludes establishment of causal relationships between the variables analyzed. In addition, relevant factors previously associated with fatigue, such as sleep quality, were not assessed. Objective measurements of PA and sedentary time were not performed, as these variables were evaluated using self-reported instruments. Moreover, stratified analysis may have been limited by the small number of participants in certain subgroups, particularly those with active disease or severe fatigue, users of specific dietary supplements, or participants reporting avoidance of particular foods. Finally, patient selection was restricted to individuals followed at a Digestive Day Hospital and receiving biological and/or immunosuppressive therapy, potentially limiting the generalizability of the findings to the broader IBD population.

## 5 Conclusions

A high prevalence of fatigue was observed among IBD patients, including those in clinical remission. Identification of intensity-specific associations between PA and fatigue levels represents a novel contribution to our understanding of fatigue in IBD. Vigorous PA appeared to be specifically reduced in fatigued patients, whereas walking and moderate-intensity activities were relatively preserved, at least in patients with mild or moderate fatigue. These findings highlight the importance of considering the intensity of physical activity when assessing fatigue in patients with IBD and provide a rationale for future longitudinal and interventional studies. Furthermore, fatigue was associated with longer sedentary time, a finding that has been infrequently reported in IBD populations. Future studies should investigate whether reducing sedentary behavior and promoting appropriate levels of physical activity may improve fatigue in this population.

## Data Availability

The raw data supporting the conclusions of this article will be made available by the authors, without undue reservation.
