# Supplementary material for: High prevalence of fatigue in patients with inflammatory bowel disease: association with physical activity intensity and sedentary behavior
Source: Front Public Health. 2026 Aug 27;14:1893458. doi: 10.3389/fpubh.2026.1893458 (PMC13558227; doi:10.3389/fpubh.2026.1893458)
Supplement: Supplementary file 1 [file Data_Sheet_1.docx]

Supplementary Material

**Supplementary Table 1.** Lifestyles and food avoidances in participants stratified per gender

|  | **All**  (n=422) | **Women**  (n=214) | **Men**  (n=208) | **p** |
| --- | --- | --- | --- | --- |
| **Alcohol consumption** |  |  |  | <0.001* |
| Never | 233 (55.2) | 133 (62.4) | 100 (48.1) |  |
| Once per week | 103 (24.4) | 54 (24.9) | 49 (23.6) |  |
| 2-3 times/week | 50 (11.8) | 17 (8.0) | 33 (15.9) |  |
| 4-5 times/week | 14 (3.3) | 1 (0.5) | 13 (6.3) |  |
| 6-7 times/week | 22 (5.2) | 9 (4.2) | 13 (6.3) |  |
| **Smoking** |  |  |  | 0.599 |
| Non-smokers | 184 (43.6) | 95 (44.4) | 89 (42.8) |  |
| Ex smokers | 156 (37.0) | 75 (35.1) | 81 (38.9) |  |
| Occasional smokers | 19 (4.5) | 8 (3.7) | 11 (5.3) |  |
| Daily smokers | 63 (14.9) | 36 (16.8) | 27 (13.0) |  |
| **Supplements** | 84 (19.9) | 45 (21.0) | 39 (18.8) | 0.558 |
| **Food avoidance** | 237 (56.2) | 136 (63.6) | 101 (48.6) | 0.002* |

Values are the mean ± SD or number (percentage) of participants; *p<0.05 indicates significant differences between genders (student t test for unpaired data or chi-square test)

**Supplementary Table 2.** Association of fatigue levels with characteristics of participants

|  | **No fatigue**  (n=126) | **Mild fatigue**  (n=184) | **Moderate fatigue**  (n=83) | **Severe fatigue**  (n=29) | p |
| --- | --- | --- | --- | --- | --- |
| **Age** (years) | 43.5 ± 15.5 | 45.6 ± 15.2 | 44.8 ± 15.1 | 46.2 ± 10.9 | 0.618 |
| **BMI** |  |  |  |  | 0.284 |
| Underweight (%) | 3 (2.4) | 7 (3.8) | 6 (7.2) | 2 (6.9) |  |
| Normal weight (%) | 67 (53.2) | 101 (54.9) | 35 (42.2) | 15 (51.7) |  |
| Overweight (%) | 46 (36.5) | 50 (27.2) | 29 (34.9) | 8 (27.6) |  |
| Obesity (%) | 10 (7.9) | 26 (14.1) | 13 (15.7) | 4 (13.8) |  |
| **IBD** |  |  |  |  | 0.851 |
| Crohn’s disease | 78 (28.7) | 118 (43.4) | 56 (20.6) | 20 (7.4) |  |
| Ulcerative colitis | 48 (32.0) | 66 (44.0) | 27 (18.0) | 9 (6.0) |  |
| **Disease scores** |  |  |  |  |  |
| Crohn’s disease (Harvey) | 0.58 ± 1.34 | 0.78 ± 1.44 | 1.36 ± 2.42 | 2.00 ± 2.22 | 0.003* |
| Ulcerative colitis (Mayo) | 0.46 ± 1.11 | 0.67 ± 1.31 | 1.19 ± 2.13 | 1.63 ± 1.77 | 0.062 |
| **Disease levels** |  |  |  |  | 0.369 |
| Remission | 114 (31.1) | 159 (43.3) | 71 (19.3) | 23 (6.3) |  |
| Active levels | 12 (21.8) | 25 (45.5) | 12 (21.8) | 6 (10.9) |  |
| **Previous IBD related surgery** | 18 (14.3) | 37 (20.1) | 22 (26.5) | 8 (27.6) | 0.158 |
| **Depression / anxiety** | 1 (0.8) | 10 (5.4) | 7 (8.4) | 3 (10.3) | 0.035* |
| **Other diseases** | 38 (30.2) | 67 (36.4) | 27 (32.5) | 15 (51.7) | 0.154 |
| **Blood parameters** |  |  |  |  |  |
| Haemoglobin (g/dL) | 14.5 ± 1.5 | 13.8 ± 1.6 | 13.7 ± 1.3 | 14.2 ± 3.4 | 0.002* |
| Iron (μg/dL) | 89.4 ± 35.4 | 81.7 ± 35.6 | 80.0 ± 38.0 | 63.3 ± 27.0 | 0.004* |
| Ferritin (ng/mL) | 91.9 ± 96.8 | 95.5 ± 131.7 | 105.9 ± 163.5 | 110.0 ± 147.8 | 0.836 |

Values are expressed as mean ± standard deviation or number of participants (percentage); *p<0.05 indicates significant differences (one-way ANOVA or chi-square test); IBD: inflammatory bowel disease

**Supplementary Table 3.** Association of fatigue levels with physical activity parameters

|  | **No fatigue**  (n=126) | **Mild fatigue**  (n=184) | **Moderate fatigue**  (n=83) | **Severe fatigue**  (n=29) | p |
| --- | --- | --- | --- | --- | --- |
| **IPAQ levels** |  |  |  |  | <0.001* |
| Low (%) | 23 (18.3) | 63 (34.2) | 39 (47.0) | 19 (65.5) |  |
| Moderate (%) | 34 (27.0) | 34 (18.5) | 20 (24.1) | 8 (27.6) |  |
| High (%) | 69 (54.8) | 87 (47.3) | 24 (28.9) | 2 (6.9) |  |
|  |  |  |  |  |  |
| **Total PA** |  |  |  |  |  |
| Mets·hour/week | 43.6 ± 47.3 | 46.7 ± 68.2 | 29.8 ± 51.9 | 10.7 ± 15.8 | 0.004* |
| Sessions/week | 10.0 ± 4.4 | 9.04 ±4.47 | 7.71 ± 3.88 | 6.76 ±3.24 | <0.001* |
| Minutes/day | 222.2±188.7 | 226.6±224.0 | 175.4±174.8 | 146.6±185.0 | 0.074 |
| **Intense PA** |  |  |  |  |  |
| Sessions/week | 1.93 ± 2.19 | 1.65 ± 2.06 | 0.68 ± 1.35 | 0.24 ± 0.64 | <0.001* |
| Minutes/day | 42.3 ±49.7 | 51.0 ± 90.6 | 21.4 ± 44.9 | 6.28±17.16 | 0.001* |
| **Moderate PA** |  |  |  |  |  |
| Sessions/week | 2.66 ± 2.57 | 2.29 ± 2.33 | 2.12 ± 2.29 | 1.21 ± 1.90 | 0.025* |
| Minutes/day | 61.9 ± 81.1 | 67.1 ± 96.9 | 64.3 ± 98.5 | 27.1 ±56.1 | 0.177 |
| **Walking** |  |  |  |  |  |
| Sessions/week | 5.44 ±2.22 | 5.10 ± 2.38 | 4.92 ± 2.49 | 5.31 ± 2.42 | 0.413 |
| Minutes/day | 118.0±133.1 | 108.5±134.7 | 89.8±119.1 | 113.1±154.3 | 0.509 |
|  |  |  |  |  |  |
| **Sitting time** |  |  |  |  |  |
| Hours/day | 5.50 ± 3.04 | 6.49 ± 3.45 | 6.29 ± 3.38 | 7.24 ± 3.84 | 0.023* |

Values are the mean ± SD or percentage of participants; *p<0.05 indicates significant differences (one-way ANOVA or chi-square test). PA: physical activity.

**Supplementary Table 4.** Association of fatigue with smoking, alcohol consumption, intake of supplements and food avoidance

|  | **No fatigue**  (n=126) | **Fatigue** (n=296) | p |
| --- | --- | --- | --- |
| **Alcohol consumption** |  |  | 0.901 |
| Never | 70 (55.6) | 163 (55.1) |  |
| Once per week | 30 (23.8) | 73 (24.7) |  |
| 2-3 times/week | 13 (10.3) | 37 (12.5) |  |
| 4-5 times/week | 5 (4.0) | 9 (3.0) |  |
| 6-7 times/week | 8 (6.3) | 14 (4.7) |  |
| **Smoking** |  |  | 0.092 |
| Non-smokers | 63 (50.0) | 121 (40.9) |  |
| Ex smokers | 47 (37.3) | 109 (36.8) |  |
| Occasional smokers | 5 (4.0) | 14 (4.7) |  |
| Daily smokers | 11 (8.7) | 52 (17.6) |  |
| **Supplements** | 16 (12.7) | 68 (23.0) | 0.016* |
| **Food avoidance** | 54 (42.9) | 183 (61.8) | <0.001* |

Values are reported as the number (percentage) of participants; p<0.05 indicates significant differences (chi-square test)
