# Supplementary material for: High prevalence of fatigue in patients with inflammatory bowel disease: association with physical activity intensity and sedentary behavior
Source: Front Public Health. 2026 Aug 27;14:1893458. doi: 10.3389/fpubh.2026.1893458 (PMC13558227; doi:10.3389/fpubh.2026.1893458)
Supplement: Supplementary file 2 [file Data_Sheet_2.docx]

**PROGRAMA DE ACTIVIDAD FÍSICA SOBRE LOS NIVELES DE FATIGA EN EII**

**PABID 1**

**Código:**

**Fecha visita:**

**Firma CI:** Sí ◻

**Variables sociodemográficas**

1. **Sexo:**

| Mujer |  |
| --- | --- |
| Hombre |  |
| No binario |  |

2. **Estado civil: C** ◻      **S** ◻        D  ◻       V ◻

**¿Con quién vive usted?**

◻ **Solo**

◻ **Con mi pareja**

◻ **Con mi familia**

3. **Lugar de residencia:**
4. **Edad:** años
5. **Talla**	cm
6. **Peso**	kg
7. **IMC:** kg/m^2^
8. **Nivel de estudios:**

| Sin educación formal |  |
| --- | --- |
| Educación primaria completa |  |
| Educación secundaria incompleta |  |
| Educación secundaria completa |  |
| Bachillerato |  |
| Educación terciaria (universitaria) incompleta |  |
| Educación terciaria (universitaria) completa |  |
| Educación terciaria (no universitaria) incompleta |  |
| Educación terciaria (no universitaria) completa |  |

9. **¿Estudia actualmente?** Sí __	No ___
10. **¿Trabaja actualmente?**	Sí __	No ___

**Cargo u ocupación:**

11. **Ingresos mensuales netos (aprox.):**

◻ <1.000€

◻ Entre 1.000-2.000€

◻ Entre 2.000-3.000€

◻ >3.000€

**Estilo de vida**

12. **Actividad física**

**Realizar IPAQ cuestionario Excel (ver drive): _________**

CUESTIONARIO INTERNACIONAL DE ACTIVIDAD FISICA (IPAQ)

Piense en las **actividades intensas** que usted ha realizado **últimamente de forma habitual** (durante el último mes). Las actividades físicas **intensas** se refieren a aquellas que implican un esfuerzo físico intenso y que lo hacen respirar mucho más intensamente que lo normal. Piense *solo* en aquellas actividades físicas que realizó durante por lo menos **10 minutos** seguidos.

-¿En cuántos **días por semana** realizó actividades físicas **intensas** tales como levantar pesos pesados, cavar, hacer ejercicios aeróbicos o andar rápido en bicicleta?

-Los días que realizó esta actividad física **intensa**, ¿cuánto tiempo dedicó a realizarla?

Piense en las **actividades moderadas** que usted ha realizado **últimamente de forma habitual** (durante el último mes). Las actividades moderadas son aquellas que requieren un esfuerzo físico moderado que lo hace respirar algo más intensamente que lo normal. Piense *solo* en aquellas actividades físicas que realizó durante por lo menos 10 minutos seguidos.

-¿En cuántos **días por semana** hizo actividades físicas **moderadas** como transportar pesos livianos, andar en bicicleta a velocidad regular o jugar dobles de tenis? **No** incluya caminar.

-Los días que realizó esta actividad física **moderada**, ¿cuánto tiempo dedicó a realizarla?

Piense en el tiempo que usted dedicó a **caminar** **últimamente de forma habitual** (durante el último mes). Esto incluye caminar en el trabajo o en la casa, para trasladarse de un lugar a otro, o cualquier otra caminata que usted podría hacer solamente para la recreación, el deporte, el ejercicio o el ocio.

-¿En cuántos **días por semana** **caminó** por lo menos **10 minutos** seguidos?

-Habitualmente, ¿cuánto tiempo en total dedicó a caminar en uno de esos días?

La última pregunta es acerca del tiempo que pasó usted **sentado** durante los días hábiles de los **últimos 7 días**. Esto incluye el tiempo dedicado al trabajo, en la casa, en una clase, y durante el tiempo libre. Puede incluir el tiempo que pasó sentado ante un escritorio, visitando amigos, leyendo, en coche, o sentado o recostado mirando la televisión.

-Durante los **últimos 7 días** ¿cuánto tiempo pasó **sentado** de media?

13. **Hábitos no saludables**

*Tabaco:*

- No fumo ni he fumado nunca de forma habitual.
- No fumo actualmente, pero he fumado antes.
  - Número cigarrillos al día:
  - Edad de inicio del consumo:
  - Edad de fin del consumo:
- Sí, pero no diariamente.
  - Número de cigarrillos a la semana:
  - Edad inicio consumo:
- Sí, fumo diariamente.
  - Número de cigarrillos al día:
  - Edad inicio consumo:

*Alcohol:*

| **Frecuencia de consumo:** |  | **Cantidad** |
| --- | --- | --- |
| Nunca |  |  |
| Una vez a la semana |  |  |
| 2-3 veces a la semana |  |  |
| 4-5 veces a la semana |  |  |
| 6-7 días a la semana |  |  |

14. **¿Sigue algún tipo de dieta especial?**	Sí __	No ___
    a. Mediterránea
    b. Vegana
    c. Vegetariana
    d. Sin carne
    e. Sin pescado
    f. Otra
15. **Indique qué alimentos no consume a excepción de los excluidos por su tipo de dieta**

| **Alimento** | **Causa** |
| --- | --- |
|  |  |
|  |  |
|  |  |
|  |  |
|  |  |
|  |  |
|  |  |
|  |  |

16. **Padece alguna otra enfermedad como hipertensión, diabetes mellitus, etc. Especificar:**
17. **Sigue algún otro tratamiento farmacológico distinto al pautado para tratar su EII.**

| **Fármaco** | **Dosis** | **Frecuencia** |
| --- | --- | --- |
|  |  |  |
|  |  |  |
|  |  |  |
|  |  |  |
|  |  |  |
|  |  |  |
|  |  |  |
|  |  |  |
|  |  |  |

18. **¿Consume algún suplemento no farmacológico?**

Sí __	No ___	¿Cuál/es?

**Enfermedad Inflamatoria Intestinal**

19. **Diagnóstico enfermedad:**

Fecha diagnóstico:

|  | E/L | B | P | A |
| --- | --- | --- | --- | --- |
| Colitis ulcerosa |  |  |  |  |
| Enfermedad Crohn |  |  |  |  |

E (CU): E1 proctitis, E2 izda, E3 pancolitis

L (EC): L1 Ileal, L2 Colónica, L3 Ileocólica, L4 Tract superior

P perianal

CU: B0 Remisión, B1 leve (<4 dps), B2 moderada (>5 dps mínima afectación sistémica), B3 grave (>6 dps y FC>90; Tª >37,5, Hgb <10,5 y VSG >30mm/h).

EC: B1 No estenosante ni perforante, B2 estenosante, B3 Perforante o masa abdominal, B4 perianal.

Edad al diagnóstico (A) A1 16 años o menos A2 17-40 años A3 > 40

**Realizar cuestionario Harvey Bradshow (EC)(Excel drive):___________puntuación**

**Realizar cuestionario Mayo (CU)(Excel drive):___________-puntuación**

19. **Fármacos activos:**

Mesalazina oral: No ◻	Sí ◻	Mesalazina tópica: No ◻	Sí ◻

Corticoides: No ◻	Sí ◻	Azatioprina: No ◻	Sí ◻	Metotrexate: No ◻	Sí

F. biológico: No ◻	Sí ◻ Especificar:

20. **Cirugía EII:** No ◻	Sí ◻	Especificar:

Resección ileocecal con anastomosis ileocólica	◻

Colectomía total con anastomosis ileorectal	◻

Proctocolectomía restauradora con reservorio ileoanal	◻

21. Resultados analítica sangre (imprimir)

Verificar anemia:

Hematíes (10^6^/μL)

Hemoglobina (g/dL)

Hierro (μg/dL)

Ferritina (ng/mL)

Transferrina (mg/dL)

22. **Realizar cuestionario Fatiga (ver Excel drive):__________puntuación**

◻Si Fatiga
